# Hypertension as a Metabolic Disorder and the Novel Role of the Gut

**DOI:** 10.1007/s11906-019-0964-5

**Published:** 2019-06-24

**Authors:** Masami Tanaka, Hiroshi Itoh

**Affiliations:** Division of Endocrinology, Metabolism and Nephrology, Department of Internal Medicine, Keio University School of Medicine, 35 Shinanomachi, Shinjuku-ku, Tokyo, 160-8582 Japan

**Keywords:** Hypertension, Obesity, Salt, Sympathetic nervous activity, Adipokines, Renin-angiotensin-aldosterone system, Sodium/glucose cotransporter, Gastrointestinal tract, Microbiota

## Abstract

**Purpose of Review:**

Hypertension is related to impaired metabolic homeostasis and can be regarded as a metabolic disorder. This review presents possible mechanisms by which metabolic disorders increase blood pressure (BP) and discusses the importance of the gut as a novel modulator of BP.

**Recent Findings:**

Obesity and high salt intake are major risk factors for hypertension. There is a hypothesis of “salt-induced obesity”; i.e., high salt intake may tie to obesity. Heightened sympathetic nervous system (SNS) activity, especially in the kidney and brain, increases BP in obese patients. Adipokines, including adiponectin and leptin, and renin-angiotensin-aldosterone system (RAAS) contribute to hypertension. Adiponectin induced by a high-salt diet may decrease sodium/glucose cotransporter (SGLT) 2 expression in the kidney, which results in reducing BP. High salt can change secretions of adipokines and RAAS-related components. Evidence has been accumulating linking the gastrointestinal tract to BP. Glucagon-like peptide-1 (GLP-1) and ghrelin decrease BP in both rodents and humans. The sweet taste receptor in enteroendocrine cells increases SGLT1 expression and stimulates sodium/glucose absorption. Roux-en-Y gastric bypass improves glycemic and BP control due to reducing the activity of SGLT1. Na/H exchanger isoform 3 (NHE3) increases BP by stimulating the intestinal absorption of sodium. Gastrin functions as an intestinal sodium taste sensor and inhibits NHE3 activity. Intestinal mineralocorticoid receptors also regulate sodium absorption and BP due to changing ENaC activity. Gastric sensing of sodium induces natriuresis, and gastric distension increases BP. Changes in the composition and function of gut microbiota contribute to hypertension. A high-salt/fat diet may disrupt the gut barrier, which results in systemic inflammation, insulin resistance, and increased BP. Gut microbiota regulates BP by secreting vasoactive hormones and short-chain fatty acids. BP-lowering effects of probiotics and antibiotics have been reported. Bariatric surgery improves metabolic disorders and hypertension due to increasing GLP-1 secretion, decreasing leptin secretion and SNS activity, and changing gut microbiome composition. Strategies targeting the gastrointestinal system may be therapeutic options for improving metabolic abnormalities and reducing BP in humans.

**Summary:**

SNS, brain, adipocytes, RAAS, the kidney, the gastrointestinal tract, and microbiota play important roles in regulating BP. Most notably, the gut could be a novel target for treatment of hypertension as a metabolic disorder.

## Introduction

Hypertension causes serious health problems if left uncontrolled [1]; it is the leading contributor to global disease burden [2, 3]. Hypertension often coexists with obesity, type 2 diabetes mellitus (DM), and dyslipidemia, referred to as *metabolic syndrom*e. Hypertension with versus without metabolic abnormalities is associated with higher risk for cardiovascular events. Hence, the risk stratification of hypertension is based on the number and severity of metabolic risk factors [4]. Several guidelines, including the Japanese hypertension guideline JSH 2014, for the management of high BP use obesity, metabolic syndrome, DM, and dyslipidemia for risk stratification among hypertensive patients [5]. For hypertensive individuals with several metabolic abnormalities, rapid initiation and intensification of BP-lowering therapy are strongly recommended.

The number of adults with hypertension worldwide increased from 594 million in 1975 to 1.13 billion in 2015 [6]. In parallel, the prevalence of obesity worldwide increased more than three times in men and more than twice in women during the last four decades [7]. According to the Global Burden of Disease Study conducted in 195 countries, the prevalence of obesity in 2015 was estimated to be 12% in adults [8]. With increasing body mass index (BMI), the prevalence of hypertension, DM, and dyslipidemia increases in a linear fashion [9••]. Therefore, hypertension is considered one of the metabolic disorders.

## Interrelationships Between High Salt Intake, Obesity, and Hypertension

The JSH 2014 strongly recommends that adults should consume less than 6 g of salt per day. However, salt intake is still high in Japan. The 2018 ESC/ESH guideline recommends that adults should consume less than 5 g of salt per day [10]. The 2017 AHA/ACC/ASH guideline defines optimal salt intake as consuming less than 1500 mg sodium (3.8 g salt) per day [11].

High salt intake links not only to increased BP but also to obesity, i.e., “salt-induced obesity” (Table 1). A cross-sectional study using a 7-day dietary record suggested significant associations between salt intake and sugar-sweetened soft drink consumption in children [12]. However, salt-obesity association was independent of energy intake [13]. The results suggest that salt intake may contribute to obesity. In an experimental study, mice fed on a high-salt diet had increased protein catabolism in the liver and skeletal muscle and showed increased food intake [14]. The catabolized proteins are converted into urea, which increases water reabsorption in the kidney. The observed increased appetite of mice could be a compensatory response to the energy-intensive salt-driven protein catabolism process.

**Table 1:** Salt-induced obesity: observations and possible mechanisms

Clinical/basic	Subjects	Observations	Possible mechanisms	Ref
Clinical studies	Human	Significant association between salt intake and sugar-sweetened soft drink consumption	NA	[12]
	Human	Significant association between salt intake and body fat mass	NA	[13]
Basic studies	Mice	High salt intake induces increased food intake	Increased salt-driven protein catabolism	[14]
	Mice	Salt intake activates the reductase-fructokinase pathway in the liver and hypothalamus, leading to endogenous fructose production	Increased leptin resistance	[15]
	Adipocytes	High salt increases adipogenesis/lipogenesis and inflammatory adipokines	Activation of salt-inducible kinase	[16•]

*BMI* body mass index, *NA* not available

## Metabolic Abnormalities Associated with High Blood Pressure

### Sympathetic Nervous System

Greater sympathetic nervous system (SNS) activity precedes BP elevation in experimental and human studies [17]. Greater SNS activity, especially in the kidney, contributes to hypertension [18]. Renal SNS increases sodium reabsorption and renin secretion and impairs pressure natriuresis. Greater SNS activity also contributes to metabolic disorders. In patients with metabolic syndrome, SNS is activated due to hyperinsulinemia, hyperleptinemia, activated renin-angiotensin-aldosterone system (RAAS), baroreflex dysfunction, and obstructive sleep apnea [19, 20]. High-fat and carbohydrate diets stimulate α1- and β-adrenergic peripheral receptors [21]. Baroreflex, which inhibits SNS activity in a compensatory manner when BP rises, is impaired in obese hypertensives [22]. Central SNS activation, induced by hyperactivity of leptin and the preproopiomelanocortin pathway, is also related to obesity and hypertension [23].

Increased microglial activation and neuroinflammation within the brain regions that control autonomic response contribute to hypertension [24]. SNS activity is heightened by activation of brain regions controlling autonomic function due to high-fat diet, salt, stress, and angiotensin II (AngII) [25, 26]. The paraventricular nucleus of the hypothalamus (PVN) integrates inputs from the brainstem and circumventricular organs with the rostral ventrolateral medulla and intermediolateral nucleus in the spinal cord [27]. In the presence of hypertension, neuroinflammation is evident with activated microglia and immigrating bone marrow progenitors assembled in PVN [28, 29••, 30]. Epigenetic aberration of PVN AngII type 1 receptor (AT1), caused by DNA methyltransferase 3a, contributes to salt-sensitive hypertension in rat offspring [31].

### Adipokines

Adipocytes secrete a variety of bioactive substances, referred to as adipokines. Under physiological conditions, adipocytes release anti-inflammatory adipokines including adiponectin, nitric oxide (NO), transforming growth factor (TGF)-β, and inerleukin-10, which improve insulin sensitivity and exert anti-atherosclerotic effect. However, in persons with metabolic disorders, adipocytes are hypertrophied and secrete pro-inflammatory adipokines including leptin, tumor necrosis factor-α, angiotensinogen, and interleukin-6, which aggravate insulin resistance and exert pro-atherosclerotic effect [32].

Lower plasma levels of adiponectin and leptin are associated with higher BMI [33]. Lower plasma adiponectin levels are associated with hypertension and metabolic disorders. Adiponectin decreases the expression of sodium/glucose cotransporter (SGLT) 2 in the kidney [34•]. Obesity decreases adiponectin secretion, which leads to higher SGLT2 expression in obese than in non-obese persons. In contrast, a high-salt diet activates peroxisome proliferator-activated receptor δ and adiponectin production, leading to decreased renal SGLT2 expression and BP. This compensatory mechanism is impaired in persons with diabetes [34•].

Increased circulating leptin levels are present in animals and humans with hypertension [35]. Leptin crosses the blood-brain barrier, acts on the hypothalamus, and regulates energy metabolism via decreasing appetite and increasing energy expenditure with heightened SNS [36]. High salt intake has been reported to activate the aldose reductase-fructokinase pathway [15] and produce fructose in the liver and hypothalamus. Increased fructose contributes to leptin resistance, and this, in turn, leads to hyperphagia, insulin resistance, fatty liver, obesity, and hypertension.

### Renin-Angiotensin-Aldosterone System

Obesity is associated with increased RAAS activity. RAAS exists within several organs, referred to as the tissue RAAS [37]. The brain, heart, kidney, immune cells, vasculature, and adipose tissue express all components of RAAS [38]. Adipocytes, especially intra-abdominal adipocytes, produce angiotensinogen and aldosterone. Urinary levels of aldosterone are associated with insulin resistance and are higher in overweight than in lean normotensive adults [39]. Soluble factors secreted from the adipose tissue, including complement-C1q TNF-related protein and leptin, increase aldosterone secretion from adrenocortical cells [40–42].

Salt intake may be associated with adipogenesis/lipogenesis and inflammation via increasing the expression of adipokines and the RAAS-related components, including α-adducin-1, cytochrome P450 family 11-subfamily β-2, and mineralocorticoid receptor [16•].

Reduced resting metabolic rate (RMR), which is partly determined by RAAS within the hypothalamus, is associated with obesity [43, 44]. AT1, agouti-related peptides (AgRP), and the leptin receptor (Lepr) are co-expressed on arcuate nucleus (ARC) cells in the hypothalamus. AT1 in the central nervous system increases energy expenditure, whereas AngII type 2 receptor in adipocytes reduces RMR [45, 46]; AT1 is expressed in a specific subset of AgRP neurons named SST3 [47, 48]. AT1 is activated via a variety of stimuli including leptin, high-fat diet, and deoxycorticosterone (DOCA)-salt and increases thermogenic SNS activity and RMR [49]. Thus, RAAS in the ARC may contribute to obesity-related hypertension.

## Gastrointestinal Tract and BP Regulation

**Fig. 1:**
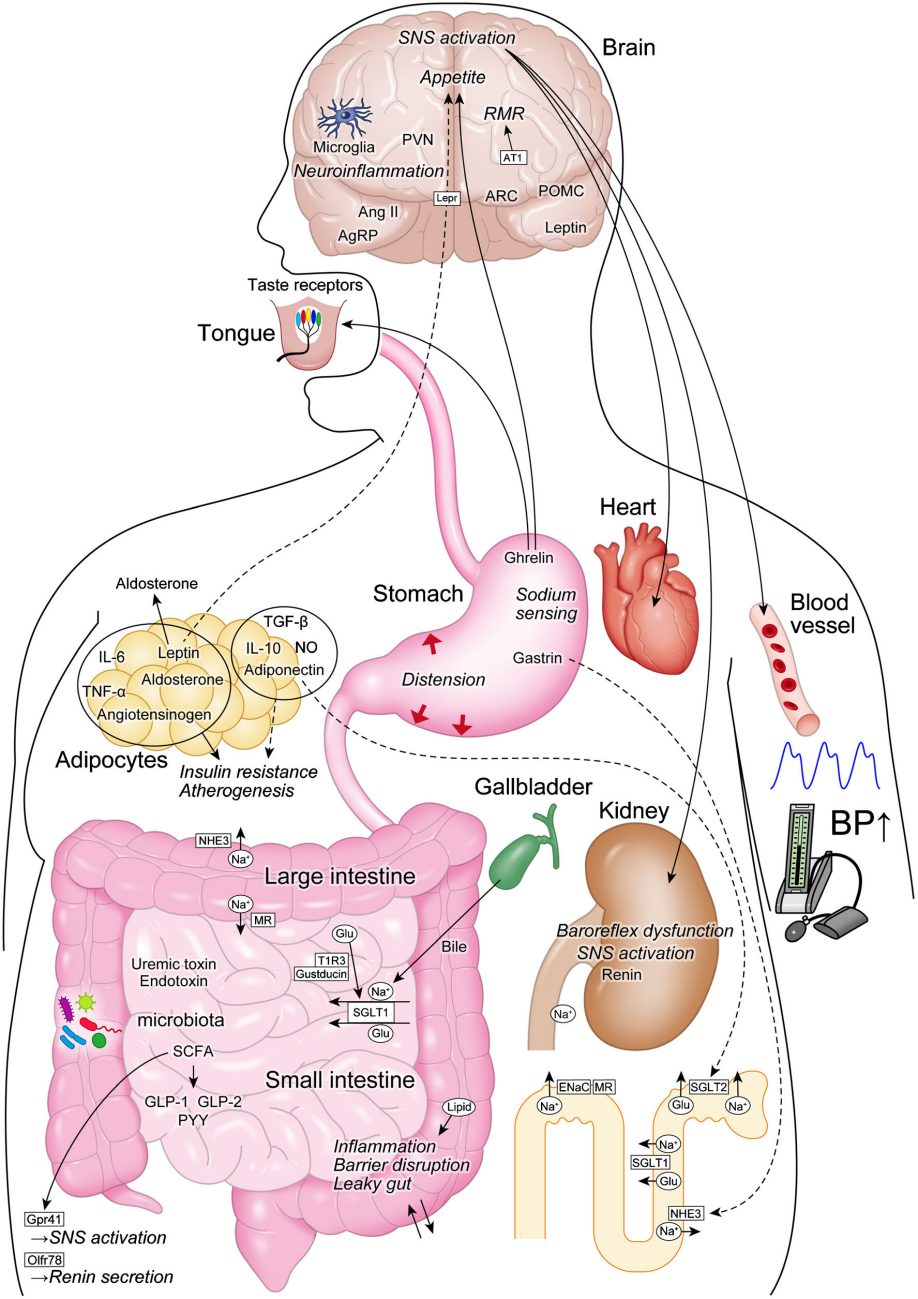
The gut, brain, and kidney play important roles in regulating blood pressure. Enhanced renal SNS induces sodium retention, increases renin secretion, and impairs pressure natriuresis. Central SNS is enhanced through increased microglial activation and neuroinflammation. Leptin acts on the hypothalamus and regulates energy metabolism by decreasing appetite and increasing energy expenditure. Adiponectin is induced by a high-salt diet and decreases the expression of SGLT2. AT1 in the brain stimulates thermogenic SNS activity, energy expenditure, and RMR. Low-pressure gastric distention raises blood pressure. Ghrelin exerts an orexigenic effect and increases taste sensitivity. Gastrin, whose secretion is stimulated by oral sodium intake, is reabsorbed by renal proximal tubules and inhibits NHE3 activity. T1R3 and gustducin act as sweet taste receptors in the intestine. When they sense sugar/sweetener, they increase the expression of SGLT1. Intestinal MR modulates ENaC activity and regulates sodium absorption. Sodium in the bile is required for the proper function of SGLT1 in the intestine. Gut microbiota produces both pro-inflammatory mediators, such as uremic toxin, and anti-inflammatory mediators, such as SCFA. SCFA stimulates the secretion of anti-inflammatory gut hormones, such as GLP-1 from the enteroendocrine cells. High-salt and high-fat diets alter the microbial composition and induce intestinal inflammation and gut barrier disruption, leading to the leaky gut mucosa. AgRP agouti-related peptide, AngII angiotensin II, ARC arcuate nucleus, AT1 angiotensin II type-1 receptor, BP blood pressure, ENaC epithelial sodium channel, GLP glucagon-like peptide, Glu glucose, IL interleukin, Lepr leptin receptor, MR mineralocorticoid receptor, Na sodium, NHE3 Na/H exchanger isoform 3, NO nitric oxide, POMC preproopiomelanocortin, PVN paraventricular nucleus, PYY peptide YY, RMR resting metabolic rate, SCFA short-chain fatty acid, SGLT sodium/glucose cotransporter, SNS sympathetic nervous system, TGF transforming growth factor, TNF tumor necrosis factor

Salt, fat, and carbohydrates are absorbed through the gastrointestinal tract, and its dysfunction causes metabolic disorders (Fig. 1). Thus, the gastrointestinal tract may be regarded as the essential organ for metabolic syndrome and hypertension [50].

### Gut Hormones

Several gut hormones contribute to vascular function and BP [51]. Chronic administration of glucagon-like peptide 1 (GLP1) improves endothelial dysfunction and reduces BP in Dahl salt-sensitive rats with a high-salt diet [52]. These effects are due to natriuretic effects of GLP1 that are attributable to changing renal hemodynamics and inhibiting Na/H exchanger isoform 3 (NHE3) activities in the renal proximal tubule. BP-lowering effects of GLP1 receptor agonists have been documented in meta-analysis of clinical trials [53].

Ghrelin, secreted mainly from the stomach, exerts an orexigenic effect including hunger sensation [54]. We demonstrated that ghrelin inhibited BP elevation and renal damage caused by AngII through an anti-oxidative stress mechanism in hypertensive mice [55]. In this study, ghrelin inhibited AngII-induced upregulations of TGF-β and plasminogen activator inhibitor-1 in the kidney, which results in ameliorating renal fibrosis. Therefore, ghrelin is a contributor to BP regulation in the context of the gastrorenal axis [56]. Furthermore, ghrelin increased the expression of mitochondrial uncoupling protein 2 (UCP2) as well as PGC1α, a key regulator of mitochondrial biosynthesis. Ghrelin administration improved physical function in sarcopenia mice via mitochondrial activation and muscular enhancement [57].

### Taste Perception

Salt taste sensitivity is related to salt consumption and thus may be associated with hypertension [58]. In Japanese women, impaired salt taste perception is associated with a higher prevalence of hypertension [59].

Taste perception is a sensory function of the gastrointestinal tract. Taste receptors are expressed on the taste buds in the tongue, the gut, and the brain [60]. Taste receptors in mammals, including the sweet taste receptor, are GTP-binding protein-coupled receptors (GPCRs). The salt taste receptor is supposed to be an epithelial sodium channel (ENaC), also called as an amiloride-sensitive sodium channel.

Luminal sugar/sweetener sensors in the intestine include the sweet-responsive type 1 taste receptor subunit 3 (T1R3) and the taste G protein gustducin. When intestinal sugar/artificial sweetener sensors are activated, SGLT1 mRNA and protein expression and glucose-absorptive capacity are increased [61•]. SGLT1 is a major pathway of transporting dietary sugars from the intestinal lumen into the enterocytes. SGLT1 is a Na^+^/glucose cotransporter, and thus sodium and glucose are absorbed concomitantly, which may result in increased BP. SGLT1 inhibitors such as phlorizin may reduce not only glucose but also BP.

Taste perception is modulated by many vasoactive and gastric hormones. The nervous system associated with the amiloride-sensitive salt taste receptors is impaired in aldosterone/sodium chloride-induced hypertensive rats [62]. AngII increases sodium intake via attenuating the sensitivity of the amiloride-sensitive salt taste receptor and increases energy intake via enhancing sweet taste sensitivity in mice [63]. Salt and lipid taste sensitivity is attenuated in ghrelin-knockout mice [64].

### Intestinal Sodium Absorption

NHE3-knockout mice have lower BP than the control mice [65]. Pharmacologic inhibition of the gut NHE3 prevents hypertension in animals and humans through inhibiting intestinal sodium absorption [66].

Gastrin is secreted from G cells in the stomach and duodenum, and its secretion is upregulated by an oral sodium intake. Gastrin is reabsorbed at the renal proximal tubules [67] and has a natriuretic effect via inhibiting renal sodium transport through the cholecystokinin type B receptor (CCKBR) on several nephron segments [68]. Gastrin inhibits the activities of NHE3 [69] and Na^+^,K^+^-ATPase [70] in renal proximal tubule cells. Gastrin inhibits sodium transport in the intestine due to stimulating cholinergic nerves or inhibiting sympathetic nerves [56]. Gastrin can be regarded as an intestinal sodium taste sensor. The CCKBR antagonist reduces natriuresis in salt-resistant mice with a high-salt diet [71]. Germline deletion of gastrin [56] and CCKBR [71] in mice reduces sodium excretion after an oral sodium load, i.e., salt-sensitive hypertension.

**Fig. 2:**
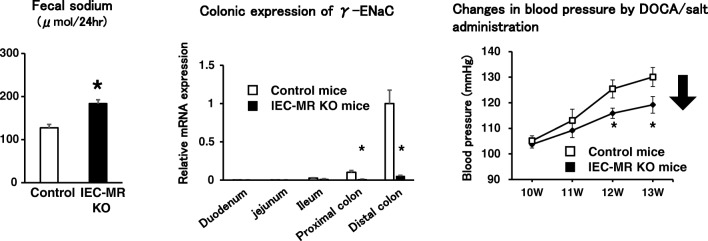
The effect of intestinal mineralocorticoid receptor knockout on fecal sodium, γ-ENaC expression, and blood pressure. *P < 0.05 versus control. Adapted from [72•]. DOCA deoxycorticosterone, ENaC epithelial sodium channel, IEC-MR KO intestinal epithelial cell-specific mineralocorticoid receptor knockout, W weeks

The mineralocorticoid receptor (MR) is expressed on the epithelial cells in the renal tubules, intestine, and skin. Renal MR regulates sodium balance through sodium absorption in the kidney. We investigated the function of MR in the intestine using intestinal epithelial cell-specific MR-knockout (IEC-MR KO) mice [72•] (Fig. 2). IEC-MR KO mice fed on a standard diet had increased fecal sodium excretion with reduced colonic expression of β- and γ-ENaC. DOCA/salt-induced BP elevation was also attenuated in IEC-MR KO mice.

Net sodium absorption in the small intestine is increased in spontaneously hypertensive rats (SHRs) [73]. The aberrant expression and function of SGLT1 in the jejunal epithelium of SHRs have been reported [74]. Baud et al. reported that the glucose uptake in the bile-deprived alimentary limb (AL) in the intestine was reduced after conducting Roux-en-Y gastric bypass (RYGB) in minipigs [75•]. When bile or sodium was added to AL, glucose uptake was restored that was blocked by the SGLT1 inhibitor phlorizin. The decrease in glucose uptake in the intestine after RYGB has been observed in humans. The glucose- and BP-lowering effects of RYGB may be attributable to reducing activity of SGLT1.

### Gastric Sodium/Volume Sensor

RYGB improved diurnal diuresis in morbidly obese patients [76]. Although patients with RYGB tend to increase salt intake, their office BPs were not elevated [77]. The observed increased salt intake in patients with RYGB may be due to a compensation for natriuresis or a change in salt appetite after the intervention. An experimental study suggested that gastric sodium sensors regulate salt appetite and have natriuretic effects in response to food and water ingestion [78]. RYGB may modify the effects of gastric sodium sensors.

Pressure-induced gastric distention increases both heart rate and BP in anesthetized rats. Gastric distension affects the splanchnic nerve systems, and this, in turn, activates the nucleus of the solitary tract, nucleus ambiguous, lateral reticular nucleus, and ventrolateral medulla [79]. The pressor response is attenuated after splanchnic denervation.

### Gut Microbiota

#### High Salt/Fat Intake and Microbiota

Changes in the composition and function of the gut microbiota contribute to cardiometabolic diseases, including hypertension, obesity, type 2 DM, and dyslipidemia [19]. When microbiota from mice with metabolic syndrome or mice genetically deficient in Toll-like receptor 5 was transferred to the germ-free wild-type mice, the wild-type mice exhibited metabolic syndrome including insulin resistance, obesity, dyslipidemia, and hypertension [80].

Most Proteobacteria and some Firmicutes family produce pro-inflammatory uremic toxins [81, 82], while Lactobacilli produce anti-inflammatory mediators, including short-chain fatty acid (SCFA) and NO [83, 84]. SCFAs increase anti-inflammatory gut hormones, including GLP-1, GLP-2, and PYY from enteroendocrine cells [83].

A high-salt diet changes the microbial composition; e.g., fecal SCFA (acetate, propionate, and isobutyrate) production is increased in Dahl salt-sensitive rats [85•]. In this study, differences in microbial composition were present between the mice fed on normal- and high-salt diets, and the abundance of seven microbial taxa was associated with BP. It was also reported that high-salt diet aggravated colitis due to changing fecal microbiota composition in mice [86•]. In the experimental murine colitis model, the relative abundance of Lactobacillus and levels of the butyrate (protective SCFA) were reduced, which results in a pro-inflammatory state in the gut.

A high-fat diet also affects the microbial composition and function [87]. A high-fat diet increases intestinal inflammation and disrupts the gut barrier, which makes the gut mucosa leaky. Bacterial endotoxins generated by gut microbiota pass the mucosal tissue and enter into the systemic circulation if the intestinal barrier was disrupted [88]. Therefore, the leaky gut mucosa induced by a fatty diet leads to inflammation [89], insulin resistance, reducing glucose uptake, and hepatic glucose production in vivo.

Using the macrophage-specific chemokine receptor 2-knockout and IEC-specific tamoxifen-inducible Ccl2-knockout mice [90••], we reported that a high-fat diet increased Ccl2 expression in the IECs that results in the recruitment of pro-inflammatory macrophages, increased gut permeability, inflammasome activation, and insulin resistance in the adipose tissue.

Oral administration of *Akkermansia (A) muciniphila* improves the gut barrier dysfunction and metabolic disorders in obese and type 2 diabetic mice [91]. Higher abundance of *A. muciniphila* is observed to be significantly associated with the improvement of cardiometabolic parameters in obese individuals undergoing caloric restriction [92]. Treatment with live or pasteurized *A. muciniphila* had no adverse effects in humans [93•], suggesting the potential novel treatment for metabolic disorders in humans.

#### Gut Microbiota and Hypertension

Emerging evidence indicates that the gut microbiota contributes to hypertension [94]. Gut microbiota generates vasoactive hormones including dopamine, norepinephrine, and serotonin [51]. Gut dysbiosis has been present in both animals [95] and humans with hypertension [96]. Firmicutes and *A. muciniphila* decrease while Bacteroidetes increases in rodents and humans with hypertension [94, 96, 97]. The ratio of Firmicutes to Bacteroidetes may be associated with hypertension.

Deletion of NHE3 changes gut microbiota composition and attenuates BP elevation in mice infused with AngII [65, 98]. SCFAs, including acetate, propionate, and butyrate, are produced by the interaction between dietary fiber and microbiota and enter into the systemic circulation. SCFAs bind to GPCRs, including Gpr41, Gpr43, Olfr78, and Gpr109a [99], and regulate BP. For example, SCFAs increase SNS activity via binding to GPR41 and increase renin secretion through Olfr78. Conversely, SCFAs induce vasodilation via binding to Gpr43 and Gpr109a [100]. Therefore, modifying gut microbiota using probiotics and antibiotics may be a possible target for regulating BP. A meta-analysis study demonstrated that probiotics reduce systolic BP by 3.56 mmHg and diastolic BP by 2.38 mmHg in 543 adults with or without hypertension [101].

Administration of minocycline changed microbiota composition and lowered BP in hypertensive rats infused with AngII [94]. Minocycline, neomycin, and vancomycin have been shown to increase systolic BP in Dahl salt-sensitive hypertensive rats. However, minocycline and vancomycin decreased while neomycin did not change systolic BP in SHR [102]. The inconsistent results suggest that the differences in genetic factors and gut microbiome may influence the effect of antibiotics on BP. Furthermore, minocycline reduces microglial activation and neuroinflammation in the brain, which improved dysbiosis and hypertension [94, 103]. In experimental studies using two hypertensive rats (rat infused with chronic low-dose AngII and SHR), the administration of CMT-3 (chemically modified tetracycline-3) into the cerebral ventricle inhibited AngII-induced activation of microglia and pro-inflammatory cytokines in the PVN of the hypothalamus [29••]. Intracerebroventricular CMT-3 administration also attenuates BP elevation and SNS activity in rats infused with AngII due to improving dysbiosis and pathological alterations (thickening of the muscular layer, increased areas of fibrosis, decreased goblet cell number, and shortening of villi) in the gut wall. These results suggest an effect of microglia and microbiota on BP, and antibiotics may be effective for controlling hypertension.

### Gastrointestinal Bypass Surgery

Gastrointestinal bypass or metabolic surgery improves metabolic abnormalities in persons with morbid obesity. Diabetes, hypertension, hyperlipidemia, and obstructive sleep apnea were substantially improved after the intervention [50]. Although BP reduction after gastrointestinal bypass surgery is attributable to weight loss, BP reduction is observed before body weight reduction [104]. Therefore, the BP-lowering effect of the surgery may be independent of weight loss. The potential mechanisms underlying the BP-lowering effects of bypass surgery include increased secretion of gut hormones, including GLP-1 and PYY [105], decreasing leptin levels [106], increasing urinary sodium excretion [107], change in gut microbiota [108], and reducing SNS activity [109].

## Conclusion

Hypertension often coexists with metabolic abnormalities. Shared underlying mechanisms between hypertension and metabolic abnormalities may be present, including altered gut hormones, intestinal sodium absorption, and gut microbiota. Strategies targeting the gastrointestinal system may be therapeutic options for improving metabolic abnormalities and reducing BP in humans.
